# Concomitant Presence of Hb Agrinio and - -Med Deletion in a Greek Male Patient with Hemoglobinopathy H: More Severe Phenotype and Literature Review

**DOI:** 10.3390/hematolrep15030050

**Published:** 2023-08-08

**Authors:** Michael D. Diamantidis, Stefania Pitsava, Omar Zayed, Ioanna Argyrakouli, Konstantinos Karapiperis, Christos Chatzoulis, Evangelos Alexiou, Achilles Manafas, Evangelos Tsangalas, Konstantinos Karakoussis

**Affiliations:** 1 Thalassemia and Sickle Cell Disease Unit, Department of Hematology, General Hospital of Larissa, 41221 Larissa, Greece; pitsavastef@gmail.com (S.P.); omar.zayed32@gmail.com (O.Z.); ioanna-arg@hotmail.com (I.A.); konstantinos.karapiperis@hotmail.com (K.K.); chatzoulis.christos@gmail.com (C.C.); manafasachilles@gmail.com (A.M.); vtsaggalas@hotmail.com (E.T.); 2 Radiology Department, General Hospital of Larissa, 41221 Larissa, Greece; dr.alexiou@me.com; 3 First Department of Internal Medicine, General Hospital of Larissa, 41221 Larissa, Greece; karakoussis@yahoo.gr

**Keywords:** hemoglobinopathy H (HbH), hemoglobin (Hb) agrinio, alpha thalassemia, severe clinical manifestations, chronic hemolysis

## Abstract

Hemoglobin (Hb) Agrinio is a rare non-deletional a-globin mutation observed almost exclusively in Greek, Spanish or other Mediterranean families. The clinical manifestations of a carrier of a single Hb Agrinio mutation (single heterozygosity) depend on the concomitant presence or absence of other mutations or variants in the beta, alpha or other modifying genes. We present a Greek patient harboring a Hb Agrinio variant plus the - -Med alpha deletional allele, having an infrequent severe form of alpha thalassemia, in contrast to the typical alpha thalassemic patient and requiring regular red blood cell (RBC) transfusions and chelation treatment. We also provide a concise literature review regarding alpha thalassemic hemoglobin variants and their molecular and clinical combinations. A phase 2, double-blind, randomized, placebo-controlled, multicenter clinical trial to determine the efficacy and safety of luspatercept (BMS-986346/ACE-536) for the treatment of anemia in adults with alpha thalassemia with the participation of our center is currently recruiting patients (NCT05664737).

## 1. Introduction

There are mainly two types of the alpha globin gene mutations. Deletional mutations (reduced (α^+^) or absent (α^0^)) are more frequently encountered and lead to the incomplete and defective production of the alpha globin chains (quantitative disorders), whereas point mutations (non-deletional) are not as common, but still exist, causing dysfunctional, irregular hemoglobin (Hb) molecules (qualitative disorders). Both of the abovementioned molecular lesions, lead to various clinical manifestations towards a distinct phenotype based on the combination of genetic defects [1]. Frequently, non-deletional mutations cause a more severe clinical syndrome, when compared with deletional molecular defects.

Clinically significant forms of alpha thalassemia are hemoglobinopathy H (HbH) disease and hydrops fetalis syndrome. HbH disease derives from the presence of only one residual functioning active alpha globin gene out of four genes. The three other remaining alpha genes are impaired. Thus, the consequent excess of beta globin chains results in moderate to severe hemolysis. There is a wide variability in clinical and hematological severity. Hypochromic anemia, hepatosplenomegaly and jaundice are the most common clinical manifestations. Usually, the anemia of HbH disease is mild without requiring regular red blood cell (RBC) transfusions, in comparison to severe transfusion dependent beta-thalassemia [1,2,3,4]. Patients with HbH disease are usually transfused in pregnancy or in rare emergency cases, like a surgery. Nevertheless, there are exceptions to this rule for HbH, especially when deletional and rare non-deletional mutations are combined to form HbH disease. Co-inheritance of unstable alpha chain variants with deletional mutations (α^0^ or α^+^ thalassemia) may also lead to a more severe than expected clinical phenotype [2,4]. In this work, focus will be given to the alpha gene mutations. However, the presence of beta gene mutations affects the clinical manifestations and modifies HbH disease phenotype, and thus these beta mutations will also be discussed in the co-existence of alpha lesions.

Hb Agrinio is considered a highly unstable Hb variant, whose hallmark is the conversion of leucine to proline at the codon 29 of the a-globin chain (CTG→CCG), which forms Hb Agrinio. It is a rare non-deletional a-globin mutation observed almost exclusively in Greek or Greek-Cypriot families. The mutated amino acid 29 is located on the beta helix of the a-globin in the B10 location. The result of this alteration is probably the reason for this highly form of unstable Hb [5,6,7]. 

It has been observed that the homozygous state for the Hb Agrinio (Hb Agrinio/Hb Agrinio, defective Agrinio variants in both alleles, albeit extremely rare) correlates with a more severe hypochromic, microcytic and hemolytic anemia, blood transfusions since infancy, low levels of HbH (<2.5%) and rare HbH inclusions [5,8,9].

Conversely, the clinical manifestations of a carrier of a single Hb Agrinio mutation (single heterozygosity) depend on the concomitant presence or absence of other mutations or variants in the beta, alpha or other modifying genes. The anemia is usually milder in this case, compared to the homozygous state [6,8,10]. 

The standard identification of an HbH disease carrier might be easily overlooked in cases with a single Hb Agrinio heterozygosity or other alpha molecular defects in one allele. These carriers can have children with HbH disease or even hydrops fetalis. The difference between the latter and the Hb Bart’s hydrops has been extensively described. Hb Bart’s hydrops are the result of a homozygous alpha thalassemia (a0-thal, alpha deletional thalassemia) with the HbF and HbA being around 65% and Hb Bart over 35% [6,8,10].

Finally, the co-inheritance of Hb Agrinio in one allele and other deletional a-thalassemia variants in the other allele (double heterozygosity state) causes a phenotype with a more severe form of HbH disease and chronic hemolytic anemia. Even though the latter is an alpha thalassemia, in this rare case, the patient will express the whole spectrum of the β-thalassemia major phenotype (regular transfusions, chelation therapy) [6,11,12]. 

## 2. Materials and Methods

We present a Greek patient harboring Hb Agrinio variant plus the - -Med alpha deletional allele, having an infrequent severe form of alpha thalassemia and requiring regular RBC transfusions and chelation treatment. We also provide a concise literature review regarding the molecular and clinical combinations of the alpha thalassemic hemoglobin variants. 

The study was conducted under the declaration of Helsinki. A written signed consent form of the patient is available and the blank form of the consent used without any identification is accessible. The Scientific Ethics Committee of the General Hospital of Larissa is responsible for the approval of the study and the identification code of the submitted project is 11858. 

## 3. Case Presentation

The male patient was born in October 2003 and was diagnosed at the age of 8 months (June 2004) due to growth retardation. DNA analysis for alpha genes (αAgrα/- -Μed) showed the concomitant presence of Hb Agrinio, a rare mutated hemoglobin form, inherited from the father (αAgrα/αα) and common - -Med alpha deletional mutation (- -Μed/αα), inherited from the mother. Both parents had normal forms of the other alpha allele, thereby they were carriers of HbH without clinical manifestations. 

Ever since, he started regular RBC transfusions. The characteristic inclusion bodies were evident inside the RBCs by microscopy. Without transfusions, the levels of Hb were as low as 6.5 gr/dL, significantly worse than the expected hematocrit for HbH. After monthly RBC transfusions, Hb initially reached 8–9 gr/dL and then was stabilized to 10 gr/dL. Chelation treatment was initiated at 2 years (June 2006) with deferasirox at a dose of 15 mg/kg with the previous oral tablet dissolved in liquid. From 2004 until late 2017, he would receive two RBC units every month, whereas since 2018 up to the present, due to the gain of height and weight, he receives three to four units every month, because of physical development (usually two units every 15 days). Since 2017 (at the age of 14), the new film-coated tablets of deferasirox have been the chelation choice of treatment, at a dose initially around 14 mg/kg/day and increasing to 17.5–21 mg/kg/day in recent years. 

The levels of lactate dehydrogenase (LDH) were increased all these years (range 676–1851 (NR 170–480 U/L)), due to excessive chronic hemolysis, caused by the combination of the unstable Hb Agrinio and the alpha mutation - -Med. Median LDH value for the last 5 years was 1188 U/L. In the last year, due to stabilization to higher levels of Hb (around 10.5 gr/dL), chronic hemolysis tends to be less and LDH is usually below 700 U/L. The main hematological and laboratory data of the patient are shown in Table 1. 

**Figure 1:**
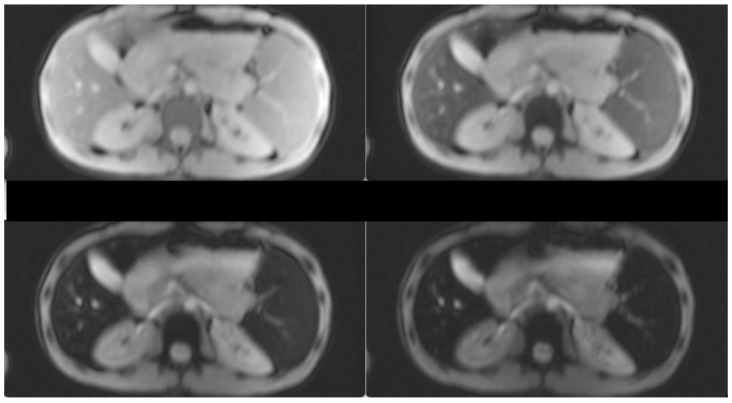
T2-star (T2*) Multi-Echo Gradient Echo images for iron quantification (the first two and the last two, out of the 12-echo images are shown). T2* Signal drop is shown in the liver and spleen at the longest echo images indicative of iron deposition. Liver T2* = 6 ms(N > 16)and LIC = 4.28 mg/gr dw (N < 1.8). Spleen T2* = 7.1 ms.

**Figure 2:**
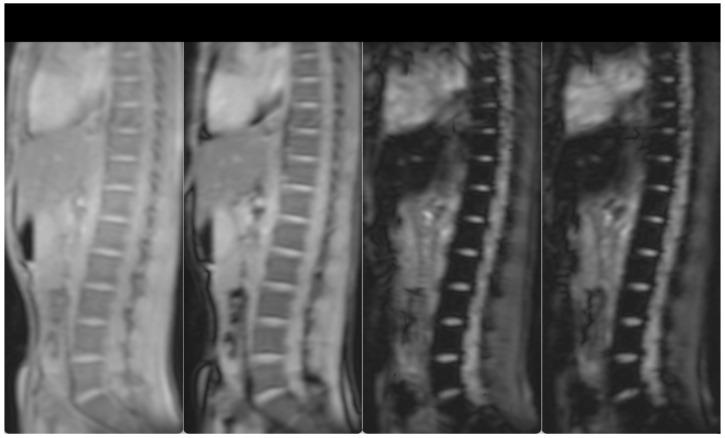
T2-star (T2*) Multi-Echo Gradient Echo images for iron quantification (the first two and the last two, out of the 12-echo images are shown). T2* Signal drop is shown in the spine bone marrow at the longest echo images indicative of iron deposition. Bone marrow T2* = 3.7 ms (N > 16).

Hepatomegaly (15 cm) and splenomegaly (18 cm) were evident, as a result of the hemoglobinopathy. Splenectomy was recommended in order to lower the transfusion burden. However, due to the lack of hypersplenism, the patient decided to increase the frequency of transfusions plus chelation treatment. After initiation of more intensive chelation, the MRI T2* showed a lack of significant iron levels both in the heart and the liver (T2* heart: 31.48 msec, T2* liver: 6.99 msec, LIC: 4.20 mg/g dw). Previous evaluations of liver iron showed mild to medium hepatic hemosiderosis, as measured by MRI T2* from 2010–2017. Hepatic, splenic and bone marrow hemosiderosis are shown in Figure 1 and Figure 2, respectively. 

Up to the present, he remains under frequent RBC transfusions (clinical similarity with homozygous beta thalassemia) with concomitant chelation with deferasirox. The Hb target is 11 mg/dL. Splenectomy has been deferred (due to the known complications of thrombotic events), and will take place only in the presence of possible hypersplenism, which is not the case until recently. 

## 4. Discussion and Literature Review

HbH in general is a mild disease. Such patients are usually transfused in pregnancy or in emergency cases, such as a surgery. Their follow-up includes an abdominal and heart ultrasound every year, along with the necessary laboratory investigations. However, the concomitant presence of Hb Agrinio plus the - -Med alpha deletional mutation, has been associated with very severe clinical manifestations, resembling these encountered in beta thalassemia major patients and requiring frequent RBC transfusions plus chelation therapy. We gave focus on the severe clinical presentation of our patient and we described the clinical evolution in time. We finally posed the dilemmas in treating the patient, especially in the era of novel therapies for thalassemia. 

Hb Agrinio was first identified in Agrinio, Greece in 1995 and named after the respective city [13]. The final clinical manifestations depend on the co-existence of this rare severe qualitative defect with other alpha or beta globin mutations. Other cases have been reported in North Macedonia [12], Cyprus [7], Spain [1,9,14] and Bulgaria [9]. A concise literature review is shown in Table 2, entitled ‘alpha thalassemic hemoglobin variants and their molecular and clinical combinations’, where the concomitant molecular defects with Hb Agrinio, along with the corresponding clinical manifestations are presented. 

Interestingly, other defective alpha hemoglobinopathies such as Hb Icaria [15] or Hb Adana [16] have been described. Three cases of compound heterozygosity for Hb Icaria plus - -Med have clinically expressed a severe form of HbH disease, which correlated with a regular need for blood transfusions from an early age, splenomegaly and growth retardation [15] (Table 2). Furthermore, the combination of Hb Adana plus the –a3.7 kb deletion (a+ thalassemia) has been associated with chronic hemolytic anemia, significant splenomegaly and thrombocytopenia [16] (Table 2). 

On the other hand, luspatercept is an agent used in the recent years for the treatment of anemia of transfusion-dependent beta thalassemia (TDT) (BELIEVE study) [17] and non-transfusion dependent beta thalassemia (NTDT) (BEYOND study) [18]. The drug inhibits Smad 2/3 signaling and enhances erythroid maturation. It exhibits its action as an activin receptor ligand trap, which prevents detrimental molecules for the RBC fate from reaching the surface of the RBC. Even though the efficacy of luspatercept is much higher in beta NTDT (thalassemia intermedia) and modest in beta TDT or beta thalassemia major, little is known regarding its value in alpha thalassemia. A phase 2, double-blind, randomized, placebo-controlled, multicenter clinical trial to determine the efficacy and safety of luspatercept (BMS-986346/ACE-536) for the treatment of anemia in adults with alpha thalassemia with the participation of our center is currently recruiting patients (NCT05664737).

In the clinical phase III trial, luspatercept was granted approval in the States and the European Union, due to the clinical finding of significantly lowering the transfusion burden by at least 33% in a series of 336 thalassemic patients [17]. It is estimated that the mechanism of action of luspatercept is not restricted to beta chains, but might involve alpha chains as well. Hence, there is molecular reasoning and there is postulation that luspatercept might work also in these rare clinical manifestations, encountered in alpha thalassemia. The forthcoming clinical trial (NCT05664737) will address the issue. Nevertheless, there is no certainty that the drug luspatercept will be effective in alpha thalassemia. It might be proven effective, but it might not. There may also be different degrees of response among individual patients and that is why the clinical trial will be conducted. The patient described has not participated in the clinical trial by the submission date of this work. He has decided to wait for the results of the trial, instead of participating.

## Figures and Tables

**Table 1:** Main hematological and biochemical laboratory data of the patient.

Hematological and Biochemical Index	
Hgb	8.1 gr/dL
WBC	4.01 k/μL
PLT	152.0 k/μL
FERRITIN	644.8 ng/mL
LDH	1821 IU/lt
TBIL	3.9 mg/dL
URIC ACID	4.1 mg/dL
UREA	52 mg/dL
CREATINE	1.0 mg/dL
GLUCOSE	111 mg/dL

**Table 2:** Alpha thalassemic hemoglobin variants and their molecular and clinical combinations.

Reference	Country	Number of Cases	Molecular Defects	Clinical Manifestations
(Galanello et al. 2011) [1] (de la Fuente-Gonzalo et al. 2012) [14]	Spain (Catalonia, Andalusia, Madrid)	14 cases (from 3 families, with the 2 of them being of Gypsy ethnicity)	a2 with mutation at codon 29 (CTG > CCG)	-11 cases of heterozygous state-silent phenotype of thalassemia without anemia and mild microcytosis with iron deficiency -3 cases of homozygous state-severe intermediate phenotype HbH disease
(Dimishkovska et al. 2017) [12]	North Macedonia	2 cases (from 2 unrelated families of Romani ethnicity)	a2 with mutation at codon 29 (CTG > CCG) [a29(B10) Leu to Pro; HBA2: c.89T > C; a^Agrinio^a/]	2 cases of homozygous state-severe or intermediate phenotype for HbH disease with blood transfusions since infancy and hemolytic anemia
(Felekis et al. 2008) [7]	Cyprus	2 cases (from 2 Greek-Cypriot families)	- -Med and Hb Agrinio [a29(B10)Leu to Pro (a2)]	2 cases with compound heterozygosity-severe or intermediateHbH disease with need for regular transfusions from early age
(Douna et al. 2008) [11]	Greece	1 case	Hb Setif, a mutation of a2-globin at codon 94, [α94(G1)Asp→Tyr, GAC > TAC (α2)], and Hb Agrinio, mutation a2-globin at codon 29, [a29(B10) Leu to Pro; CTG > CCG (a2)]	1 case with compound heterozygosity-expression of mild anemia, with no need for regular transfusions and normal development
(Szepetowski et al. 2022) [9]	-Spain (2 different families with gypsy ethnicity) -Bulgaria (1 family)	8 cases	Homozygous Hb Agrinio [a29(B10) Leu to Pro; HBA2: c.89T > C; a^Agrinio^a]	Severe or intermediate phenotype for HbH disease with blood transfusions since infancy and hemolytic anemia or even hydrops fetalis
(Traeger-Synodinos et al. 2010) [6]	Greece	-12 cases homozygous/compound heterozygous -over 25 cases of single heterozygocity (from a 15 year survey)	-Hb Agrinio mutation (α^Agrinio^α/α^Agrinio^α) -Hb Agrinio and the polyadenylation signal (polyA)site mutation (AATAAA > AATAAG, HBA2:c.*+94A > G or α^PA^α -Hb Agrinio and the α0 deletion Med -Hb Agrinio and the α2-globin gene IVS-I donor site pentanucleotide Hemoglobin deletion (HBA2:c.95 + 2_95 + 6delTGAGG or α^Hph^α) -Hb Agrinio and Hb Setif, [α94(G1)Asp→Tyr, GAC > TAC(α2) or HBA2:c.283G >T].	-4 cases of homozygous state-severe or intermediate phenotype for HbH disease with blood transfusions from early infancy and hemolytic anemia or even hydrops fetalis -8 cases of compound heterozygosity-wide range of phenotypic severity from mild thalassemia with no transfusions and mild anemia to regularly blood transfusions every 15 days, splenectomy and development deficiency
(Kanavakis et al. 1996) [15]	Greece	3 cases	- -Med/a^ICaria^a	3 cases of compound heterozygosity for Hb Icaria-clinically expressing a severe HbH disease with regular need for blood transfusions from an early age, development deficiency and splenomegaly
(Tampaki et al. 2020) [16]	Greece	1 case	Hb Adana (a^adana^a) (HBA2:c.179G>A or HBA1) (-a3.7 kb deletion/Hb Adana)	Chronic hemolytic anemia and significant splenomegaly, thrombocytopenia with no blood transfusions

## Data Availability

All data of the study are available in our department (DNA Result, laboratory results and radiological images) with the initial of the patient upon request. No identifiable information is provided for the patient, due to personal data.
